# A Role of Tomato UV-Damaged DNA Binding Protein 1 (DDB1) in Organ Size Control via an Epigenetic Manner

**DOI:** 10.1371/journal.pone.0042621

**Published:** 2012-08-21

**Authors:** Jikai Liu, Xiaofeng Tang, Lanyang Gao, Yongfeng Gao, Yuxiang Li, Shengxiong Huang, Xiaochun Sun, Min Miao, Hui Zeng, Xuefen Tian, Xiangli Niu, Lei Zheng, Jim Giovannoni, Fangming Xiao, Yongsheng Liu

**Affiliations:** 1 Ministry of Education Key Laboratory for Bio-resource and Eco-environment, State Key Laboratory of Hydraulics and Mountain River Engineering, College of Life Science, Sichuan University, Chengdu, China; 2 School of Biotechnology and Food Engineering, Hefei University of Technology, Hefei, China; 3 Department of Plant, Soil and Entomological Sciences, University of Idaho, Moscow, Idaho, United State of America; 4 United States Department of Agriculture-Agricultural Research Service, Robert Holly Center and Boyce Thompson Institute for Plant Research, Cornell University, Ithaca, New York, United State of America; National Taiwan University, Taiwan

## Abstract

Epigenetic modification generally refers to phenotypic changes by a mechanism other than changes in DNA sequence and plays a significant role in developmental processes. In this study, we found that overexpression of one alternatively spliced tomato *DDB1* transcript, *DDB1^F^* that is prevalently present in all tested tissues, resulted in reduction of organ size. Transgenic plants constitutively expressing the *DDB1^F^* from a strong cauliflower mosaic virus (CaMV) 35S promoter displayed moderately reduced size in vegetative organs (leaves and stems) and radically decreased size in reproductive organs (flowers, seeds and fruits), in which several genes encoding negative regulators for cell division were upregulated. Significantly, reduction of organ size conferred by overexpression of *DDB1^F^* transgene appears not to segregate in the subsequent generations, suggesting the phenotypic alternations are manipulated in an epigenetic manner and can be transmitted over generations. This notion was further substantiated by analysis of DNA methylation level at the *SlWEE1* gene (encoding a negative regulator of cell division), revealing a correlation between less methylation in the promoter region and elevated expression level of this gene. Thus, our results suggest DDB1 plays an important role in regulation of the epigenetic state of genes involved in organogenesis, despite the underlying mechanism remains to be elucidated.

## Introduction

The UV-damaged DNA binding protein 1 (DDB1) was originally identified as a nuclear factor that binds to UV-damaged DNA and participates in versatile DNA repair pathways at the stage of binding and recognition [1]. A growing body of evidence suggests that, conserved from yeast to human, the DDB1 acts as an adapter linking the CUL4-ROC1 catalytic core to substrate receptors to form the DDB1-CUL4-ROC1 complex that is recently identified as a cullin-RING ubiquitin ligase [2]. This DDB1-CUL4-based ubiquitin E3 ligase involves in many physiological and developmental processes, such as transcription, cell cycle, cell death and embryonic development [3], [4]. Particularly in plants, the DDB1-CUL4-based E3 ligase complex (DDB1-CUL4-RBX1) has been found to function in regulation of photomorphogenesis [5], ABA signaling [6], flowering time control [7], parental imprinting [8], UV-B tolerance and genome integrity [9], [10].

Epigenetic modifications on chromatin structure without changes in DNA sequence, in many cases through DNA methylation or/and histone modification, usually result in repressing gene expression. It has been demonstrated that epigenetic modifications play a role in various physiological processes in plants ranging from plant growth and reproduction regulation to stress responses [11], [12], [13]. In plants, the epigenetic state of a particular gene can be inherited during cell propagation, through both mitosis and meiosis. In the case of latter, the epigenetic state can be transmitted over generations, despite the mechanisms underlying this transgenerational epigenetic inheritance are still largely unknown [14]. For example, the epigenetic state of enhanced homologous recombination in *Arabidopsis* induced by stress cues can be transmitted over 4 generations [15].

It is generally thought there are two major mechanisms responsible for epigenetic modifications: DNA methylation and histone modification [13]. DNA methylation on the cyclic carbon-5 of cytosine can be asymmetric (^m^CpHpH) and symmetric (^m^CpG/^m^CpHpG) and often occurs in the promoter region, which results in repression of gene transcription [11]. One of the key questions regarding epigenetic regulations in developmental biology is how the epigenetic states are maintained faithfully through successive rounds of cell division. Recent investigations in metazoans, plants and microorganisms point to an important and conserved role of the DDB1-CUL4-containing ubiquitin E3 ligase in perpetuating epigenetic marks on chromatin, presumably via regulating the histone modification or/and DNA methylation. For example, in human, CUL4-DDB1 complex was shown to be essential for histone H3 methylation at K4 or K9 and K27, respectively, through interaction with multiple WD40-repeat proteins and Polycomb-group proteins [16], [17]. In mammalian cells, the histone H4 monomethylase PR-Set7 is shown to be a target of ubiquitin-conjugated degradation regulated by CUL4-DDB1^Cdt2^-mediated PCNA-dependent E3 ligase activity during DNA damage and replication [18], [19]. The *Arabidopsis* MSI1, a WD40 repeat protein, physically interacts with DDB1A and forms CUL4-DDB1A-MSI1 E3 ligase complex that is required to maintain *MEDEA* parental imprinting by interacting with the epigenetic regulatory *Polycomb* repressive complex 2 (PRC2) [8]. Another WD40 repeat protein, MSI4, is reported as a DDB1- and CUL4-associated factor that represses the expression of flowering locus C (*FLC*) and flowering locus T (*FT*) through its association with a CLF-Polycomb Repressive Complex 2 (PRC2) in *Arabidopsis* [7]. However, a very recent study suggests that MSI4 may not act as part of the PRC2-like complexes but act in concert to establish a repressive chromation environment at *FLC* for its transcriptional silencing [20]. Recently, several research groups have demonstrated that ubiquitin ligase components CUL4, DDB1 and DCAF (DDB1- and Cul4-associated factor) are essential for DNA methylation in the filamental fungus *Neurospora crassa* [21]–[23].

Tomato (*Solanum lycopersicum*) is an economically and experimentally important crop. Tomato fruits carrying the *high pigment 1* mutations (*hp-1* and *hp-1^w^*) are characterized by an enhanced biogenesis of plastids coupled with elevated levels of functional metabolites including carotenoids and flavonoids [24]–[27]. Previously, we have characterized the tomato *HIGH PIGMENT 1* (*HP1*) gene encoding a protein homologous to human DDB1 and two alternatively spliced transcripts have been identified without further elucidation on their function [28]. Genetic analysis suggests this *HP1*/*DDB1* plays a pivotal role in regulation of plastid division [28]–[30]. The *HP1/DDB1* is also implicated in pathogenesis-related (*PR*) gene induction and basal defense in response to non-pathogenic *Agrobacterium tumefaciens* [31]. In addition, ectopic expression study infers that the DDB1 protein may be profoundly involved in regulating a variety of developmental aspects in tomato plants [27]. Nevertheless, how DDB1 regulates these various physiological processes is largely unknown.

In the current investigation, we found a significant role of DDB1 in the organogenesis in tomato, apparently via an epigenetic regulation. Transgenic tomato overexpressing the alternatively spliced transcript of tomato *DDB1* exhibited reduction of organ size and enhanced expression of genes negatively regulating cell division probably due to less methylation. Significantly, these phenotypic alternations in both T_1_ and T_2_ generation plants were not necessarily associated with the *DDB1^F^* transgene, implicating an inheritable epigenetic state existing in these plants.

## Results

### Differential Expression Patterns of Two Alternatively Spliced DDB1 Transcripts (DDB1^F^ and DDB1^+15^)

**Figure 1:**
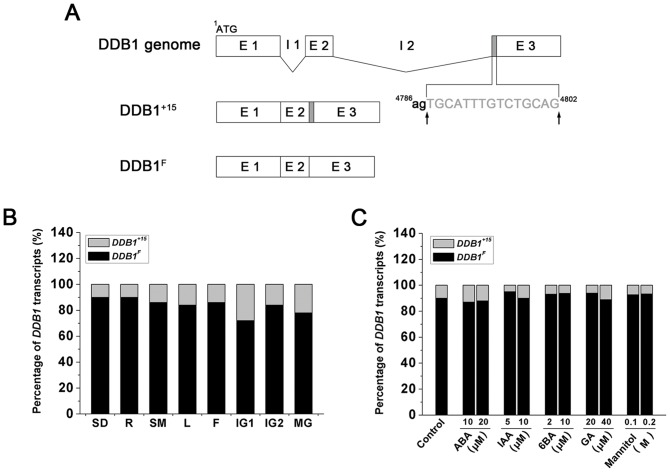
Expression analysis of transcript variants of *DDB1*. (A) Schematic diagram of *DDB1* gene splicing pattern. Introns (I) and exons (E) are marked by lowercase and uppercase letters, respectively. Numbers above the letter and arrows indicate the position of the nucleotide in the genome sequence and the site of alternative splicing, respectively. The extra 15 bp nucleotides of *DDB1^+15^* transcript are shown in grey. (B) The percentage of two *DDB1* transcripts in different tissues. SD: seedlings; R: roots; SM: stems; L: leaves; F: flowers; IG1: immature green fruits at 7-DPA; IG2: immature green fruits at 14-DPA; MG: mature green fruits at 35-DPA. The light gray and black bars indicate the expression percentage of the *DDB1^+15^* and *DDB1^F^* transcripts, respectively. Data presented are the means of three technical replicates. Similar results were obtained in at least two independent experiments. (C) Identification of the percentage of two *DDB1* transcripts in seedlings under hormones or stress treatment. Total RNAs were extracted from 10-day-old seedlings with application of abscisic acid (ABA), auxin (indole acetic acid; IAA), cytokinin (6-benzylaminopurine, BA), gibberellins (GA) and mannitol. 10-day-old seedlings without any hormone and stress treatment were used as control. The light gray and black bars indicate the expression percentage of the *DDB1^+15^* and *DDB1^F^* transcripts, respectively.

In eukaryotes, a large number of precursor mRNAs with introns can undergo alternative splicing (AS) to produce structurally and functionally different proteins from the same gene [32]. Previously, two different transcripts derived from *HIGH PIGMENT 1* locus, designated as *DDB1^F^* and *DDB1^+15^*, respectively, (GenBank accession No. AY531660 and AY531661) were identified [28]. The ORF (open reading frame) of *DDB1^+15^* contains additional 15 bp nucleotides (tgcatttgtctgcag) compared to that of *DDB1^F^* (Figure 1A). To determine the differential tissue-specific expression of the two alternatively spliced transcripts *DDB1^F^* and *DDB1^+15^*, we performed a detailed analysis of transcript composition and expression ratio in seedlings, roots, stems, leaves, flowers and fruits at different developmental stages by using a polymerase chain reaction (PCR)-based approach (described in detail in Materials and Methods). As shown in Figure 1B, in all the tissues tested, two transcripts were differentially accumulated: about 70–90% of the detected *DDB1* transcripts were the isoform of *DDB1^F^*, suggesting *DDB1^F^* isoform is prevalently expressed in tomato plants.

To further investigate the possible effect of phytohormone and stress cues on the alternative splicing of the *DDB1* transcripts, 10-day-old tomato seedlings were treated with various phytohormones or subjected to abiotic stress, including four phytohormones [abscisic acid (ABA), auxin (indole acetic acid; IAA), cytokinin (6-benzylaminopurine, BA), and gibberellins (GA)] and the stressing chemical mannitol. As shown in Figure 1C, no significant change was observed in untreated or treated seedlings and the *DDB1^F^* was still the dominant transcript.

### Overexpression of DDB1^F^ Isoform Affects Organ Size in an Epigenetic Manner

**Figure 2:**
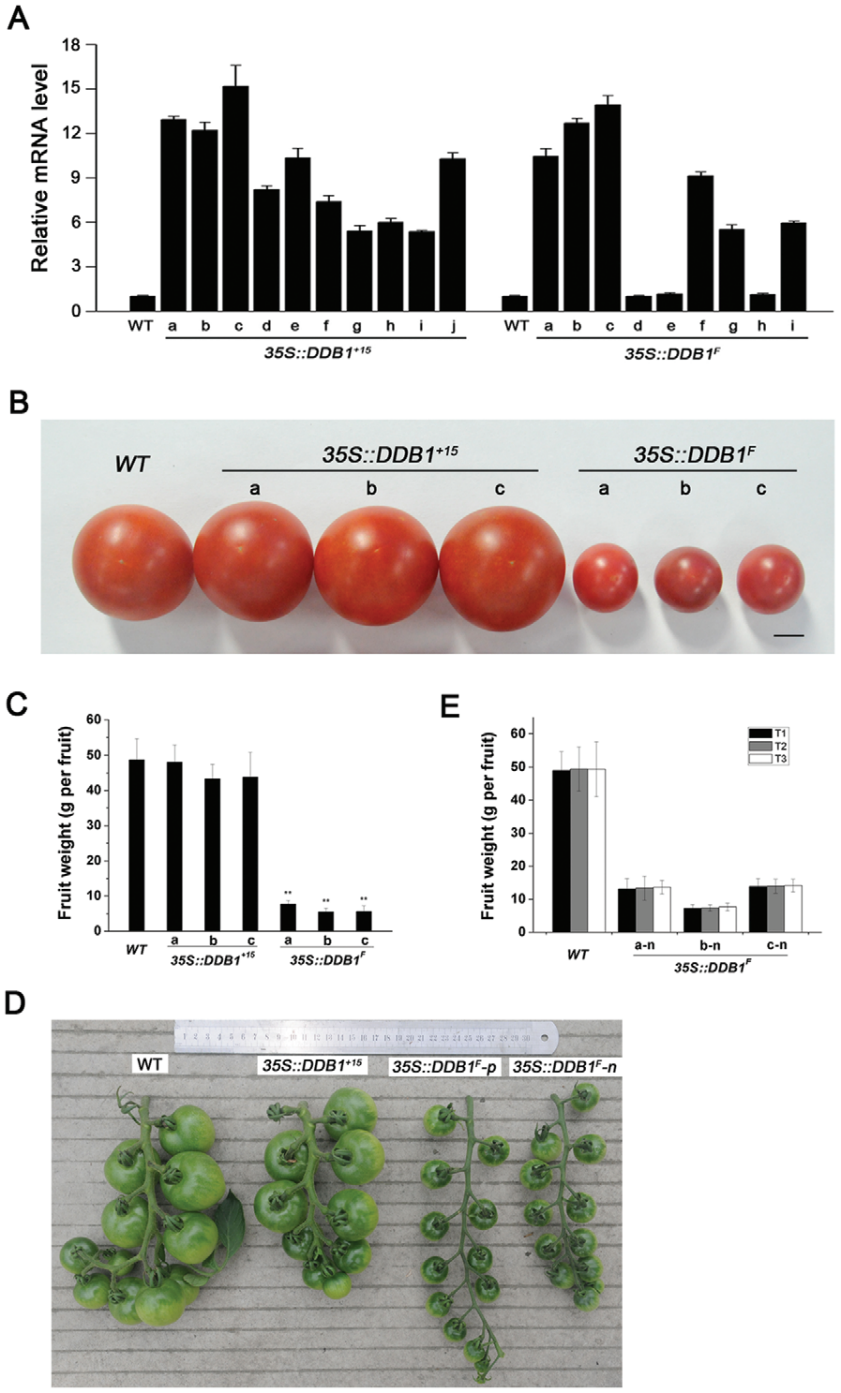
*DDB1* mRNA level analysis and phenotypes in transgenic plants. (A) Real-time PCR analysis of total *DDB1* mRNA level in WT Ailsa Craig (*WT*), *35S::DDB1^+15^* and *35S::DDB1^F^* T_0_ transgenic lines. a–j and a–i stand for 10 and 9 independent transgenic lines for *35S::DDB1^+15^* and *35S::DDB1^F^* constructs, respectively. Each bar represents three repetitions from each RNA sample (derived from pools of at least three fruits per plant). Error bars representing standard errors are shown in each case. (B) Representative red ripe fruits from field-grown plants of WT Ailsa Craig (*WT*) and 3 independent T_0_ transgenic lines (a, b and c) containing *35S::DDB1^+15^* or *35S::DDB1^F^*. Bar = 1 cm. (C) Comparison of red ripe fruit weight from WT Ailsa Craig (*WT*) and 3 independent T_0_ transgenic lines (a, b and c) containing *35S::DDB1^+15^* or *35S::DDB1^F^*. Mean values from 10 fruits from each line and standard errors are shown. Statistical analysis was performed using Student's *t*-test. (*P<0.05, **P<0.01). (D) Mature green fruits from WT Ailsa Craig (*WT*), *35S::DDB1^+15^* and *35S::DDB1^F^* T_1_ transgenic lines. (E) Comparison of red ripe fruit weight of T_1_, T_2_ and T_3_ nullizygous *35S::DDB1^F^* plants segregated out from 3 T_0_ transgenic lines (lines a, b and c). Mean values from 10 fruits from each line and standard errors are shown. Error bars representing standard errors are shown in each case. p and n represent plants with or without transgene, respectively.

The differential expression level of two alternative *DDB1* transcript isoforms prompted us to investigate the biological significance of *DDB1^F^* or *DDB1^+15^* transcript by using gain-of-function strategy. We generated transgenic tomato overexpressing *DDB1^F^* or *DDB1^+15^* under the control of the constitutive CaMV 35S promoter. 9 and 10 primary transgenic T_0_ lines harboring *35S::DDB1^F^* or *35S::DDB1^+15^* were created and verified by PCR using NPTII (Kan^r^)-specific primers. All 10 *35S::DDB1^+15^* lines (named *35S::DDB1^+15^* a to j) were morphologically indistinguishable from the nontransformed wild type (WT) plants despite the overexpression of *35S::DDB1^+15^* transgene was verified by real-time RT-PCR analysis, whereas 6 of 9 *35S::DDB1^F^* lines displayed significantly small fruits, which weighed only 11–16% of the nontransformed WT fruits (Figure 2A, B, C). Further real-time RT-PCR assay indicated the *35S::DDB1^F^* transcripts were dramatically overaccumulated in these 6 lines producing small fruits (named *35S::DDB1^F^* a, b, c, f, g and i) but not in the other 3 lines (Figure 2A), suggesting the phenotype of reduced size fruits is caused by overexpression of *35S::DDB1^F^*. Since the morphological appearance of T_0_ transgenic lines may be affected by the tissue culture process, we followed up the observed phenotype of *35S::DDB1^F^* transgenic lines to T_1_ generation by self-pollination. To our surprise, the small fruit phenotype caused by *35S::DDB1^F^* did not segregate with the *35S::DDB1^F^* transgene. All T_1_ plants derived from the 6 lines of *35S::DDB1^F^* transgenic plants produced small fruits (Figure 2D). Subsequently, we selected T_1_ plants carrying hemizygous *35S::DDB1^F^* transgene derived from 3 T_0_ lines (lines a, b and c) to generate T_2_ generation. As expected, the *35S::DDB1^F^* transgene exhibited a 3∶1 segregation ratio in T_2_ population, and all plants produced small fruits regardless harboring *35S::DDB1^F^* transgene or not. In the mean time, we also selected nullizygous (without *35S::DDB1^F^* transgene) T_1_ plants to generate the T_2_ and T_3_ nullizygous *35S::DDB1^F^* tomato plants. As expected, no significant alteration in fruit size was observed among T_1_, T_2_ and T_3_ fruits (Figure 2E). Thus, we conclude: firstly, the small fruit phenotype elicited by overexpression of *35S::DDB1^F^* transgene can be regulated in an epigenetic manner; secondly, this epigenetic state can be inherited over generations.

### The Altered Organogenesis Is Characterized by Substantial Reduction in Organ Size

**Figure 3:**
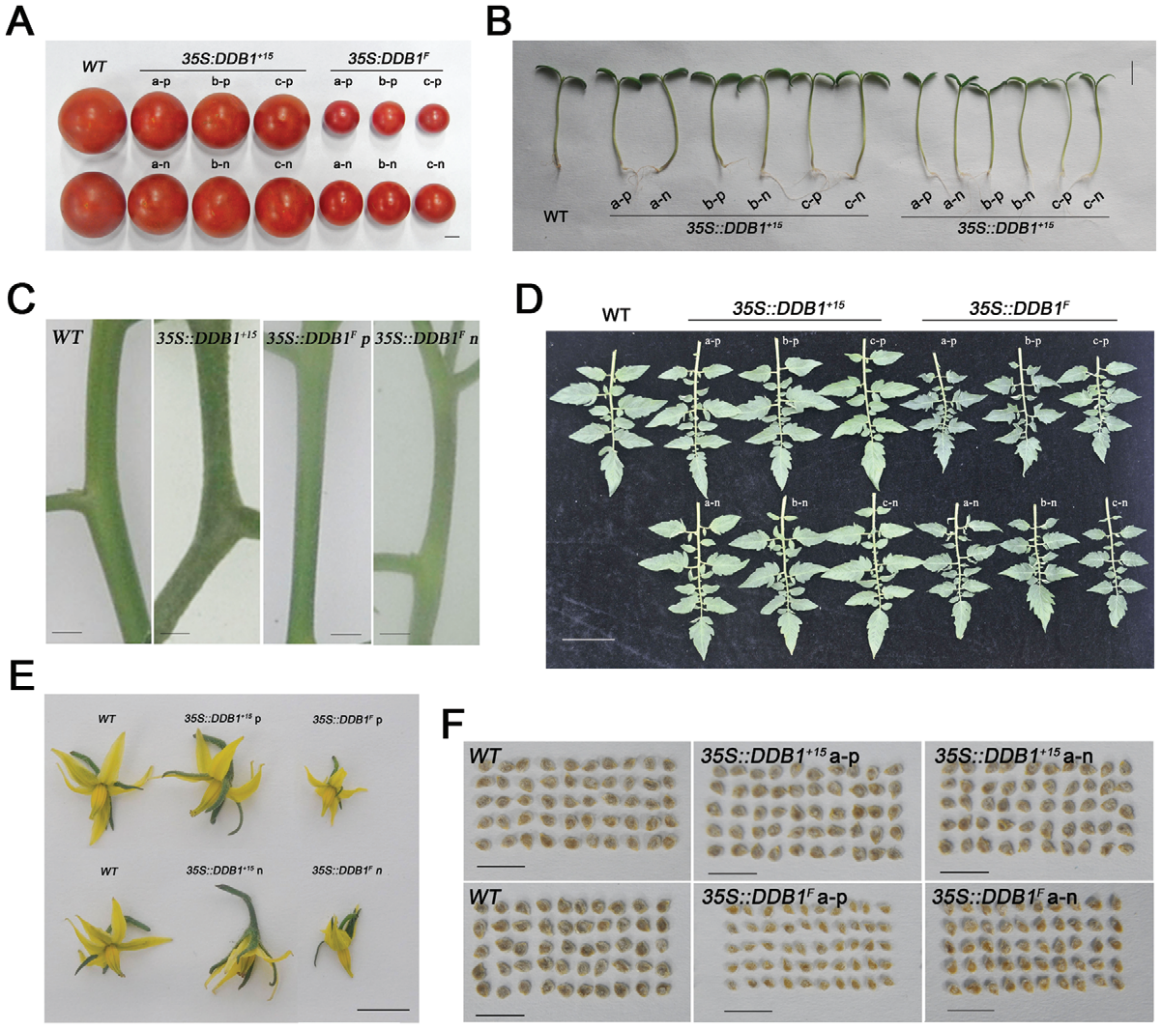
Organ size reduction in *DDB1^F^* overexpression line. (A) Representative red ripe fruits from field-grown plants of WT Ailsa Craig (*WT*), *35S::DDB1^+15^* and *35S::DDB1^F^* T_2_ transgenic lines (a, b, c). Bar = 1 cm. (B) Representative 7-day-old seedlings of WT Ailsa Craig (*WT*), *35S::DDB1^+15^* and *35S::DDB1^F^* T_2_ transgenic lines (a, b, c) germinated under white-light (16 h light/8 h dark photoperiod). Bar = 1 cm. (C) Comparison of stems from WT Ailsa Craig (*WT*), *35S::DDB1^+15^* and *35S::DDB1^F^* T_2_ transgenic lines, showing the reduction in stem size. Bar = 1 cm. (D) Comparison of mature leaves from WT Ailsa Craig (*WT*), *35S::DDB1^+15^* and *35S::DDB1^F^* T_2_ transgenic lines (a, b, c). Bar = 5 cm. (E) Comparison of flowers from WT Ailsa Craig (*WT*), *35S::DDB1^+15^* and *35S::DDB1^F^* T_2_ transgenic lines. Bar = 1 cm. (F) Comparison of seeds from WT Ailsa Craig (*WT*), *35S::DDB1^+15^* and *35S::DDB1^F^* transgenic lines. p and n represent plants with or without transgene, respectively. Bar = 1 cm.

The reduced size of fruits in the *35S::DDB1^F^* transgenic plants was the morphological change that caught our attention at first. To investigate other possible altered morphological features in the progeny of the *35S::DDB1^F^* transgenic plants, we examined the T_2_ generation plants displaying small fruits with or without *35S::DDB1^F^* transgene more closely. As shown in Figure 3, besides reduced size in fruits (Figure 3A), a significant size reduction occurred in all the observed organs including seedling roots and stems (Figure 3B), stems (Figure 3C), mature leaves (Figure 3D), flowers (Figure 3E) and seeds (Figure 3F). In contrast, there's no significant alteration in organ/tissue size observed in T_2_ generation plants derived from transgenic overexpressing *DDB1^+15^* driven by CaMV 35S promoter (Figure 3A, B, C, D, E, F).

**Figure 4:**
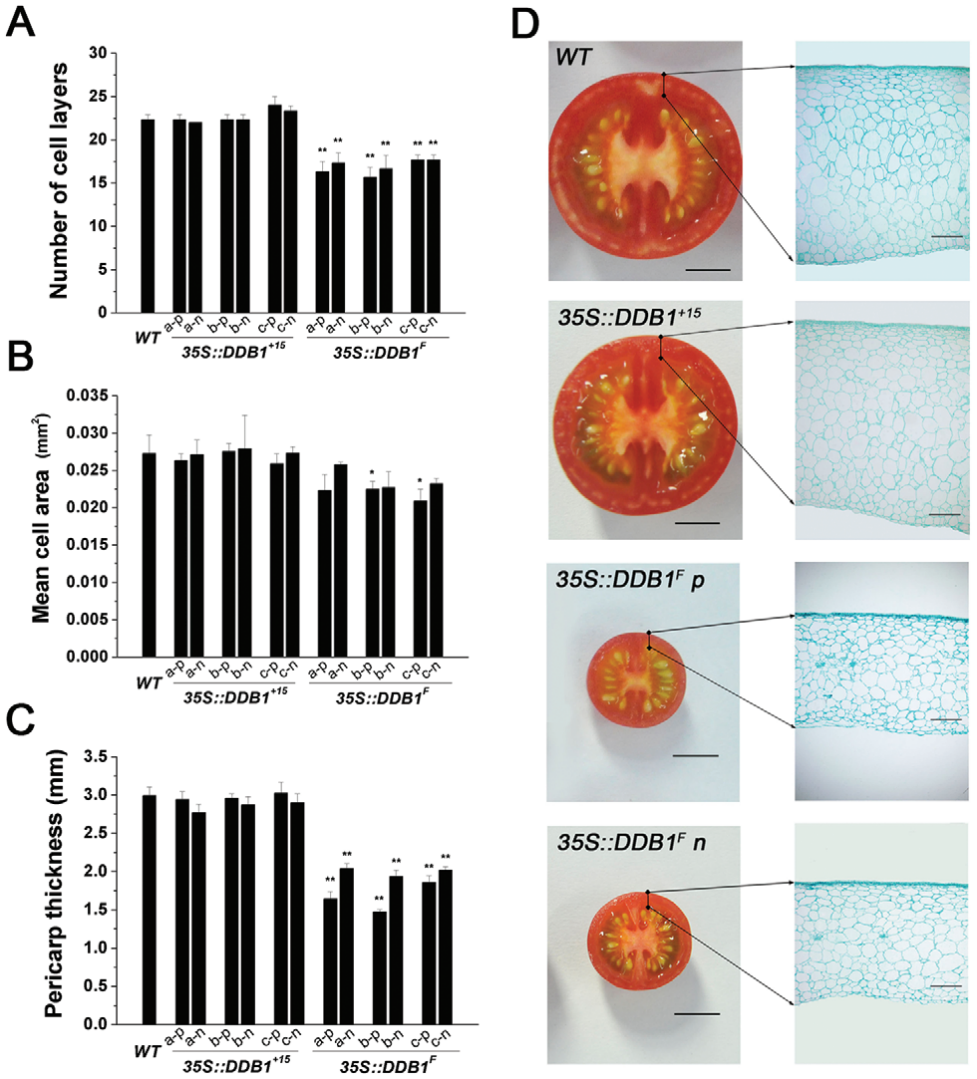
Cellular parameters of pericarp of fruits at breaker stage from WT Ailsa Craig (*WT*) and *DDB1* overexpression lines. (A) Number of cell layers across pericarp. (B) Mean cross-sectional area of one pericarp cell. (C) Pericarp thickness. (D) Cross section of fruit (left) and pericarp (right) are shown for WT Ailsa Craig (*WT*), *35S::DDB1^+15^* and *35S::DDB1^F^* T_2_ transgenic lines. p and n represent plants with or without transgene, respectively. Bar: 1 cm (F, left sections), 500 µm (F, right sections).

As both cell size and cell number could affect final organ size, we next sought to investigate the cytological basis of the drastically altered organ size, focusing on the fruit pericarp tissue due to a sharp reduction observed in fruit size. Paraffin sections were generated and then histochemically stained with fast green using pericarp tissues harvested from the T_2_ small-fruited plants (with or without *35S::DDB1^F^* transgene) or non-transgenic WT plants at breaker stage. As shown in Figure 4, at the breaker stage wherein the cell division and cell expansion ceased completely, the pericarp tissue of small fruits was significantly thinner and displayed an incisive decrease of cell layers, while the cell size was slightly reduced as compared with that of the non-transgenic WT plants. These observations suggest that overexpression of the *DDB1^F^* isoform has influence on cell division.

### Reduction of Organ Size Is Correlated With the Enhanced Expression of Genes Encoding Negative Regulators of Mitotic Division

**Figure 5:**
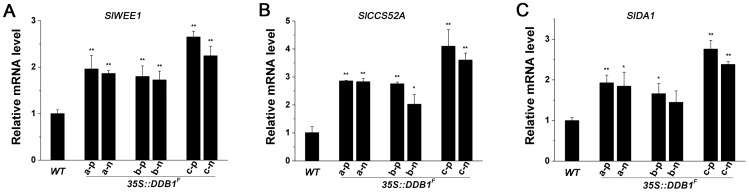
Real-time RT-PCR analysis of mRNA levels of genes regulating cell division in 7-DPA fruits from WT Ailsa Craig (*WT*) and *35S::DDB1^F^* T_2_ transgenic lines (a, b, c). p and n represent plants with or without transgene, respectively. Each bar represents three repetitions from each RNA sample (derived from pools of at least three fruits per plant). Error bars representing standard errors are shown in each case. Statistical analysis was performed using Student's *t*-test (*P<0.05, **P<0.01). Similar results were obtained in at least two independent experiments.

It has been demonstrated in recent publications that several negative cell-division regulators are involved in controlling meristem size and final organ size in plants, including *WEE1*, *CCS52A* and *DA1* [33]–[37]. It is possible that the reduction of organ size originally elicited by overexpression of *DDB1^F^* is implemented through regulating these genes. To test this hypothesis, fruit tissues from the small-fruited T_2_ plants with or without *35S::DDB1^F^* transgene or from non-transgenic WT plants were harvested at cell division stage (7-DAP) for total RNA extraction. Gene-specific primers were designed to determine the expression of tomato orthologs of these 3 genes, *SlWEE1* (GenBank accession No. AM180939.1), *SlCCS52A* (SGN-U570015) and *SlDA1* (SGN-U584698). Real-time PCR analysis using the gene-specific primers revealed the transcription of all the 3 tested genes were significantly enhanced in fruit tissues from the small-fruited T_2_ plants, regardless of containing *35S::DDB1^F^* transgene or not, using *SlUBI3* (GenBank accession No. X58253) as internal control (Figure 5). We also performed real-time PCR using *SlGAPDH* (GenBank accession No. U97257.1) as the second reference gene, and obtained the similar results (Figure S1). Thus, our results suggest the reduced organ size phenotype is probably due to upregulation of genes involved in cell-division and, significantly, this is not dependent on the original cue – the *35S::DDB1^F^* transgene.

### Correlation Between Elevated Expression of the SlWEE1 Gene and Less Methylation in Its Promoter Region

**Figure 6:**
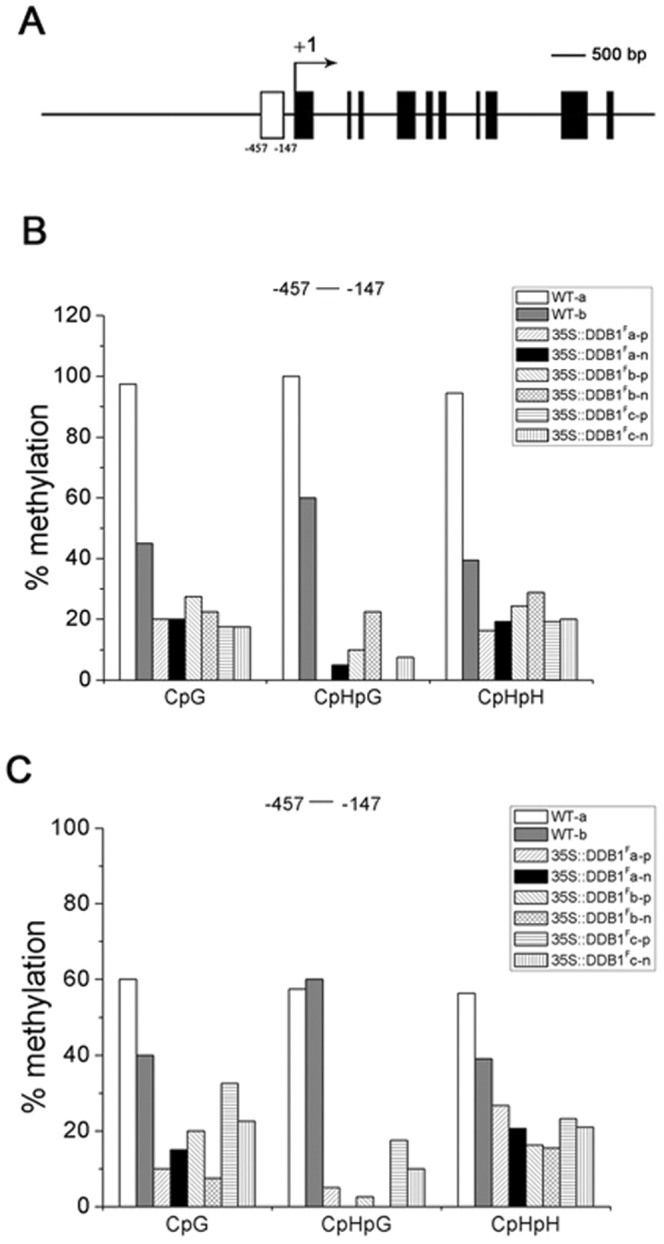
Analysis of *SlWEE1* methylation through bisulfite genomic sequencing. (A) A schematic diagram of *SlWEE1* gene structure, with +1 indicating the transcription start site and rectangle representing the methylation region −457 to −147. (B) and (C) Histograms of the percentage of CpG, CpHpG, and CpHpH methylation at region −457 to −147 of the *SlWEE1* gene from WT Ailsa Craig (*WT*) and *35S::DDB1^F^* T_1_ (B) and T_2_ (C) transgenic lines (a, b, c). p and n represent plants with or without transgene, respectively. H represents A, T or C.

It has been demonstrated that DDB1 acts as a CUL4-based ubiquitin E3 ligase component and plays a pivotal role in regulation of chromatin compaction during cell division by recruiting enzymes involved in histone methylation for ubiquitin/proteasome-mediated degradation [38], [39]. In addition, there is a growing body of evidence indicating histone modification is a prerequisite for DNA methylation and consequently represses gene expression [40]. We thus predicted that overexpression of *DDB1^F^* would affect above genes via regulation of their methylation states and this epigenetic regulation itself could be implemented via an epigenetic manner, in another word, may indirectly impacting methylation of genes of the transgenic progenies not containing the *35S::DDB1^F^* transgene. To test this notion, we examined the correlation between the enhanced *SlWEE1* expression and its possibly reduced DNA methylation during the cell division stage of fruit development. Using the approach of bisulfite genomic sequencing, DNA methylation levels of the promoter region of the *SlWEE1* gene (genomic sequences of −457 to −147 bp upstream the start codon) were determined between the immature fruits (7-DPA) of nontransgenic WT plants and both T_1_ and T_2_ plants with reduction of organ size derived from three individual primary *35S::DDB1^F^* transgenic lines. As shown in Figure 6, in fruits from the T_1_ progenies with or without the *35S::DDB1^F^* transgene, both CpG dinucleotide motifs and non-CG motifs in the tested region of the *SlWEE1* genomic sequences displayed less methylation level than in fruits from nontransgenic WT plants (Figure 6B). Similar result was also found in T_2_ progenies (Figure 6C). These results not only indicate a correlation between enhanced expression of the *SlWEE1* and decreased methylation in its promoter region, but also suggest that, at least in part, the reduced organ size phenotype is possibly due to upregulation of genes controlling cell division via epigenetic demethylation, which is originally triggered by overexpression of *DDB1^F^* transgene.

## Discussion

One of the key questions in developmental biology is how epigenetic states of gene activity are maintained faithfully through successive rounds of cell division. Recent investigations in metazoans, plants and microorganisms point to an important and conserved role of the CUL4-DDB1-containing ubiquitin ligase in perpetuating epigenetic marks on chromatin [7], [16], [18], [19], [22]. Thus, it is reasonable to hypothesize that genetic manipulation of *DDB1* gene, either by knock-down or up-regulation, may result in impact on the epigenetic state of certain genes. In this study, we have demonstrated that overexpression of *DDB1^F^* isoform, but not *DDB1^+15^* isoform, can significantly affect the organogenesis in tomato plants, apparently via an epigenetic manner.

### DDB1^F^ Is the Prevalent Form and Plays a Role in Organogenesis

We have previously showed that tomato *DDB1* gene possesses two alternatively spliced transcripts [28], designated as *DDB1^F^* and *DDB1^+15^*, respectively. In the current investigation, we demonstrate the *DDB1^F^* isoform accounts for the overwhelming majority of the expressed *DDB1* mRNAs in all the tissues tested (Figure 1B). Unlike many other examples where alternative splicing is regulated by hormones and other abiotic stress [41], the ratio of these two transcripts remains largely unchanged in response to 4 different hormones and mannitol, again suggesting the *DDB1^F^* isoform plays a dominant role in the physiological processes in tomato plants. This hypothesis is further supported by our data from experiments in which overexpression of *DDB1^F^*, but not *DDB1^+15^*, exhibited reduced-size organs, including fruits, seedling roots and stems, stems, leaves, flowers and seeds (Figure 3A, B, C, D, E, F). These results are consistent with a recent observation that overexpression of the *DDB1* gene from the CaMV 35S promoter in transgenic tomato does not cause morphological changes [42]. Nevertheless, although our results do not provide much information on the physiological significance of *DDB1^+15^* transcript, we cannot rule out the possible role of *DDB1^+15^* transcript in other physiological processes. Further experiments such as specific knockdown of *DDB1^+15^* transcript will be helpful to determine its function.

### Correlation Between Reduction of Organ Size and Enhanced Expression of Genes Encoding Negative Regulators of Cell-Division

Overexpression of the *DDB1^F^* resulted in reduced size of many organs, which is supported by the histological analysis showing that the decreased thickness of fruit pericarp is mainly due to the attenuation of cell division as evident from the reduced cell layers and cell numbers (Figure 4). This is consistent with observation of slightly faster cell division rate in *hp1* mutant plants in response to cytokinin [43]. Moreover, in T_2_ generation plants derived from the primary *35S::DDB1^F^* transgenic lines, correlation was found between reduction of organ size and enhanced expression of several genes encoding negative regulators of cell-division (*SlWEE1*, *SlCCS52A* and *SlDA1*) (Figure 5). It is possible that the upregulation of these negative regulator genes leads to premature termination of the mitotic division due to more production of the encoded proteins, resulting in proliferative growth arrest in organs. In fact, both WEE1, a kinase partially responsible for the functional abrogation of CDK/CYC complex whose activity is required for mitotic division, and CCS52A, acting as a substrate-specific activator of the anaphase-promoting complex (APC) ubiquitin ligase, are involved in the transition of mitotic cycles to endoreduplication cycles [37], [44]–[46]. Increases in either WEE1 or CCS52A activity result in inhibition of mitosis and reduced cell number, but still allow DNA replication to occur with cell enlargement as a consequence of endoreduplication [36], [37]. Thus, further investigation of the possible altered enzyme activity of WEE1 in the transgenic tomato plants will be helpful to understand the complexity of these cell division-related factors, which will be one of the topics of our future research. Nevertheless, since the final organ size, determined by cell number and size, must be compromised by these two counteracting cellular processes, it is contradictory but understandable that altering tomato *CCS52A* (*SlCCS52A*) expression in either a negative or positive manner would result in reduction of fruit size [37]. The expression levels of several mitotic-associated genes, including *CDKB* (RefSeq: NM_001246970.1), *CYCB2* (RefSeq: NM_001246857.1) and *CYCA* (RefSeq: NM_001246833.1) were also determined. But there was no significant alterations observed between WT plants and transgenic plants (Figure S2), suggesting the specificity of DDB1 effect on genes involved in regulation of cell division.

### Inheritable Epigenetic State Initiated by Overexpression of DDB1^F^ Is Not Dependent on the 35S::DDB1^F^ Transgene

Based on the observation that reduction of organ size phenotype did not segregate in T_1_ population (containing homozygous, hemizygous or nullizygous *35S::DDB1^F^* allele), in which the *35S::DDB1^F^* transgene must show segregation, we conclude that this unique phenotype of reduction of organ size is controlled by an epigenetic manner. By the same token, T_2_ plants (without harboring *35S::DDB1^F^* transgene) exhibiting organ size reduction were found not necessarily associated with the *35S::DDB1^F^* transgene, also suggesting this epigenetically phenotypic change can be transmitted over generations.

We speculate that overexpression of *35S::DDB1^F^* could cause epigenetic change on certain genes and this epigenetic alternation is inheritable. The DNA methylation analysis on the *SlWEE1* promoter region suggests the overexpression of *35S::DDB1^F^* probably leads to decrease of DNA methylation level. However, how DDB1^F^ involves in manipulation of DNA methylation is largely unknown. Recently, several research groups have demonstrated that ubiquitin ligase components CUL4, DDB1 and DCAF (DDB1- and Cul4-associated factor), are essential for DNA methylation in the filamentous fungus *Neurospora crassa* [21]–[23]. Genetic analysis by impairing components of the E3 complex suggests that the CUL4-DDB1 E3 ubiquitin ligase indirectly regulates DNA methylation via histone H3K9 trimethylation [22]. Coimmunoprecipitation-based biochemical analysis also showed CUL4-DDB1 ligase recruits histone methyltransferase DIM-5 for histone H3K9 trimethylation [21]. Moreover, interaction between CUL4-DDB1 ligase components and the gene-specific chromatin was shown to donate epigenetic repression or parental imprinting of target genes by associations with the epigenetic regulatory Polycomb Repressive Complex 2 in *Arabidopsis* [7], [8]. All these observations point to a conserved positive role for DDB1 protein in maintenance of DNA methylation and/or repressed gene activities. However, in both human cells and *Xenopus* egg extract, biochemical and molecular evidence demonstrated that Set8 protein, a methyltransferase that monomethylates histone H4 on lysine 20 (H4K20me1) [47], [48], is targeted for proteolysis in S phase during cell division by the CUL4-DDB1 E3 ubiquitin ligase [38], [39]. Similarly, in our tomato system, it is possible that DDB1-CUL4 E3 ligase manipulates cellular factors involved in methylation of genes (such as *SlWEE1*) via ubiquitination-mediated proteasome-dependent degradation. These factors likely are positive regulators of DNA methylation, such as enzymes involved in methylation of histones or factors negatively regulating the DNA demethylation. In the tomato system, overexpression of *DDB1^F^* may result in expediting the degradation of these positive regulators, consequently ablating the methylation reaction of target genes for their up-regulation. Nevertheless, the decreased methylation of the *SlWEE1* gene is apparently initiated by, but not dependent on, the *35S::DDB1^F^* transgene, despite the underlying mechanism remains to be elucidated, which will be the subject of our future research.

## Materials and Methods

### Plant Growth Conditions

Tomato plants were germinated and grown in the greenhouse under standard conditions (26°C day, 18°C night; 16 h light, 8 h dark). Primary transformants (T_0_), transgenic generation 1 and 2 (T_1_ and T_2_) plants were planted in the greenhouse and transplanted into the field 35 days later.

### Analysis of DDB1 Transcripts

Total RNAs were extracted from seedlings, roots, stems, leaves, flowers and fruits pericarps at various developmental stages (7, 14, 35-DPA). The ratio of alternatively spliced forms of *DDB1* mRNA was evaluated by PCR-based analysis. Briefly, the first-strand cDNA prepared from these organs/tissues was used in RT-PCR. Gene-specific primer set (forward primer: 5′-CATTCCTGTGTTGGCAATTTC-3′; reverse primer: 5′-AGATTATTTTGAGCCCATGGC-3′) was used to amplify both *DDB1^+15^* and *DDB1^F^*. The RT-PCR products containing mixed fragments derived from either *DDB1^F^* or *DDB1^+15^* were cloned into the pGM-T vector and subjected to a second PCR reaction to verify the identity of *DDB1* isoform and quantify the original amount of mRNA of *DDB1^F^* or *DDB1^+15^*. This second PCR was conducted using an alternative splicing-specific primer set (forward, 5′- GGTTTACCAGTGCATTTGTCTG-3′; reverse, 5′-AGATTATTTTGAGCCCATGGC-3′) that specifically target *DDB1^+15^*,but not *DDB1^F^* without containing the extra 15 bp nucleotides, and to unambiguously distinguish *DDB1^+15^* from *DDB1^F^*. The accuracy of this second PCR method was confirmed by the subsequent sequencing analysis for the randomly selected clones.

### Plasmid Construction and Tomato Transformation

DNA manipulations were carried out by using standard procedures [49]. Sequences from *DDB1^F^* and *DDB1^+15^* cDNA (accession No. AY531660 and AY531661) for construction of overexpression vectors were amplified from proper plasmids by PCR with primers DDB1OE-F (5′-GGATCCATGAGTGTATGGAACTACGTGG-3′), DDB1OE-R (5′-GAATTCCTAATGCAACCTTGTCAACTC-3′). The fragments were inserted into vector PBI121 (driven by the 35S promoter) at the *BamH*I and *EcoR*I restriction enzyme sites. Then, NOS terminator fragment from PBI121 vector was inserted into *EcoR*I restriction site by PCR using primers NOS-F (5′-GAATTCGCTCGAATTTCCCCGATC-3′), NOS-R (5′-AGTGAATTCCCGATCTAGTAAC-3′). The direction of insertion was confirmed by sequencing.

Transgenic plants were generated by *Agrobacterium tumefaciens*-mediated transformation according to the method described by Fillatti *et al.* (1987) [50], and transformed lines were first selected for kanamycin (70 mg l^−1^) resistance and then analyzed by PCR to determine the presence of T-DNA. The primers designed to the NPTII (Kan^r^) marker of PBI121 for confirmation of integration were 5′-ATTGAACAAGATGGATTGCACG-3′ and 5′-CTCGTCAAGAAGGCGATAGAAG -3′.

### Molecular Analyses

Total RNAs were extracted using Trizol reagent according to the protocol provided by the manufacturer (Invitrogen, http://www.Invitrogen.com/) and treated with DNaseI (TaKaRa; http://www.takara-bio.com). Primers for real-time RT-PCR were designed for *DDB1* (DDB1 rt-F, 5′-CCGAGAATTGCAGACAGAATGT-3′; DDB1 rt-R, 5′-CCCTCTTCATGCTTGAAAATCA-3′), *SlWEE1* (WEE1 rt-F, 5′-GAGCAAATCGGTAGTGGGAAC-3′; WEE1 rt-R, 5′-CCATCAAAGCCTGTCTCCTATC-3′), *SlCCS52A* (CCS52A rt-F, 5′-TTCAGGAAGCCGAGACAAGAG-3′; CCS52A rt-R, 5′-AAGCCGATTATCATTTCCACCT-3′), *SlDA1* (DA1 rt-F, 5′-TTTCAATGTCAGATAACCGTCCA-3′; DA1 rt-R, 5′-AATTAGACCAGCCGGATTTGTT-3′), and the control *SlUBI3* (UBI3 rt-F, 5′-AGGTTGATGACACTGGAAAGGTT-3′; UBI3 rt-R, 5′-AATCGCCTCCAGCCTTGTTGTA-3′), *SlGAPDH* (GAPDH rt-F, 5′-AGGACTGGAGAGGTGGAAGAGC-3′; GAPDH rt-R, 5′-CGACAACGGAGACATCAGCAGT-3′). The real-time PCR was performed using an SsoFast EvaGreen Supermix (Bio-rad catalog #172-5203). Each sample was amplified in triplicate and all PCR reactions were performed on the Applied Biosystems StepOne Real-Time PCR System (Applied biosystems, http://www.appliedbiosystems.com.cn/). Dissociation curve analysis was performed at the end of each run to ensure that unique products were amplified. The tomato *SlUBI3* gene was used as an internal reference. The RT-PCR conditions were as follows: 95°C for 30 s, followed by 40 cycles of 95°C for 5 s, 60°C for 20 s. The expression level was normalized to the *SlUBI3* control, and relative expression values were determined against the buffer-treated sample or the WT Ailsa Craig (*WT*) sample using the 2^−ΔΔCt^ method. To confirm the specificity of the PCR reaction, PCR products were verified on a 1% agarose gel for the accurate amplification product size.

### Phenotypic and Cytological Analysis

After fruit ripening, fruits from individual plants were collected and weighed. At least 10 fruits from individual plants were measured. After weighing, fruits were transversally cut in half and imaged.

Cytological analysis was performed according to the methods described previously [51]. Fruits at breaker stage of WT Ailsa Craig and transgenic lines were prepared for cytological analysis by a paraffin-embedding method. An equatorial slice was excised and cut into fragments less than 4 mm wide before immersion in the fixative. During fixation, a partial vacuum was applied to extract intercellular gas. Samples were rinsed, dehydrated through an ethanol series, and embedded in paraffin. Sections (10 µm thick) were made with LEICA RM2235 microtome, stained with fast green, and photographed on OLYMPUS BX51 microscope with an OLYMPUS DP71 camera.

### Bisulfite Sequencing

Bisulfite sequencing was performed according to the previously described methods [52]. 4 µg of genomic DNA was heated at 94°C for 5 min, and quenched on ice. 5.5 µL NaOH (3 M; freshly prepared) was added and incubated at 42°C for 30 min, followed by the addition of 416 µL of bisulfite solution to the denatured DNA. Bisulfite solution was prepared as follows: 3.76 g of sodium bisulfite (Fisher S654-500) was dissolved in 4 mL of water, adjusted to pH 5.0, 330 µL of 20 mM hydroquinone was added (Sigma-Aldrich H-9003), and the volume was adjusted to 10 mL water. Samples were incubated in a PCR machine for five cycles of 55°C for 3 h and 95°C for 5 min. After bisulfite conversion, the universal DNA Purification Kit was u sed to remove extra salt (Tiangen). Then, NaOH was added to a final concentration of 0.3 M and incubated at 37°C for 20 min. 28 µL 5 M ammonium acetate was added to the bisulfite-treated DNA. Finally, the bisulfite-treated DNA was precipitated with 3 volumes of ethanol. DNA was dissolved in 50 µL of water. Semi-nested PCR was performed, and the PCR products were cloned into pGM-T vector (Tiangen) and sequenced. The primers used for *SlWEE1* were WEE1-577F (5′-TGTtATTTGGTTGTGAATAAGTTAT-3′), WEE1-457F (5′-TTATTATGATATGAGTGGTGGAA-3′) and WEE1-147R (5′-AAAATTTCATATATAACAAACTTATTAC-3′), with lowercase letters representing C-to-T or G-to-A substitution in *SlWEE1*, respectively.

## Supporting Information

Figure S1 **Real-time RT-PCR analysis of mRNA levels of genes regulating cell division using** ***SlGAPDH*** **as the reference gene.** Real-time RT-PCR analysis of mRNA levels of genes regulating cell division in 7-DPA fruits from WT Ailsa Craig (WT) and *35S::DDB1^F^* T_2_ transgenic lines (a, b, c). p and n represent plants with or without transgene, respectively. Each bar represents three repetitions from each RNA sample (derived from pools of at least three fruits per plant). Error bars representing standard errors are shown in each case. Statistical analysis was performed using Student's t-test (*P<0.05, **P<0.01).
(TIF)

Figure S2 **Semi-quantitative RT-PCR analysis of mRNA levels of** ***SlWEE1, SlCDKB, SlCYCB2*** **and** ***SlCYCA*** **genes at different fruit development stages.** Semi-quantitative RT-PCR analysis of mRNA levels of *SlWEE1* gene and cell cycle controlling genes in 7-DPA, 21-DPA and 35-DPA fruits from WT Ailsa Craig (WT) and *35S::DDB1^F^* T_2_ transgenic lines (a, b, c). p and n represent plants with or without transgene, respectively. Since the expression level of each gene was reduced with the fruit development, we used different PCR cycles at each stage. 7-DPA: 25 cycles; 21-DPA: 28 cycles; 35-DPA: 32 cycles.
(TIF)
