# Supplementary figures and images for: A Role of Tomato UV-Damaged DNA Binding Protein 1 (DDB1) in Organ Size Control via an Epigenetic Manner
Source: PLoS One. 2012 Aug 21;7(8):e42621. doi: 10.1371/journal.pone.0042621 (PMC3424292; doi:10.1371/journal.pone.0042621)

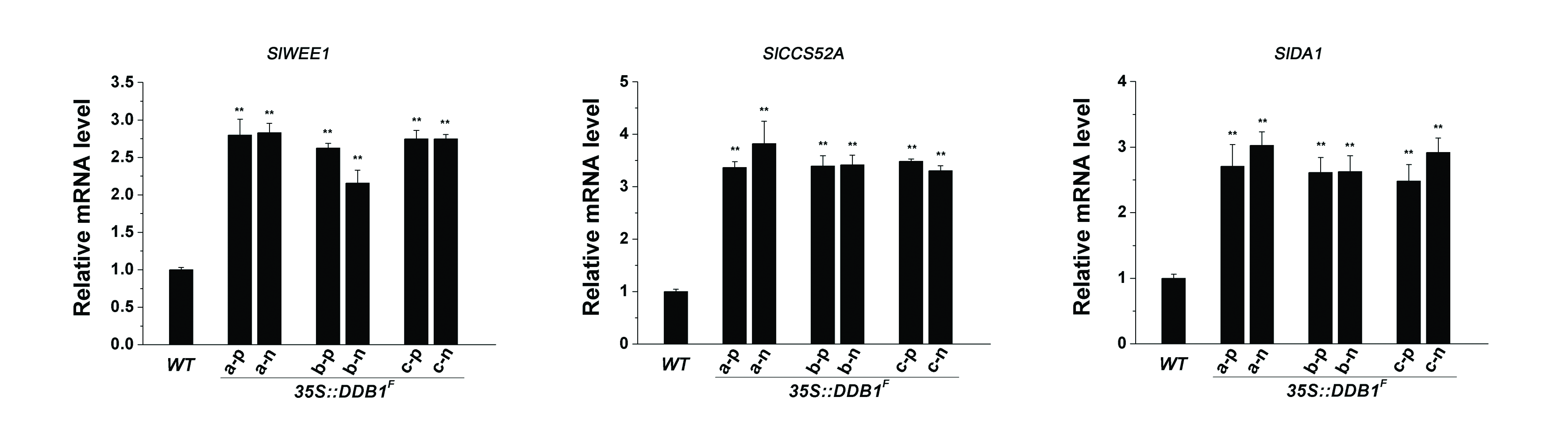

Supplement: Figure S1 — Real-time RT-PCR analysis of mRNA levels of genes regulating cell division using SlGAPDH as the reference gene. Real-time RT-PCR analysis of mRNA levels of genes regulating cell division in 7-DPA fruits from WT Ailsa Craig (WT) and 35S::DDB1F T2 transgenic lines (a, b, c). p and n represent plants with or without transgene, respectively. Each bar represents three repetitions from each RNA sample (derived from pools of at least three fruits per plant). Error bars representing standard errors are shown in each case. Statistical analysis was performed using Student's t-test (*P<0.05, **P<0.01). (TIF) [file pone.0042621.s001.tif]

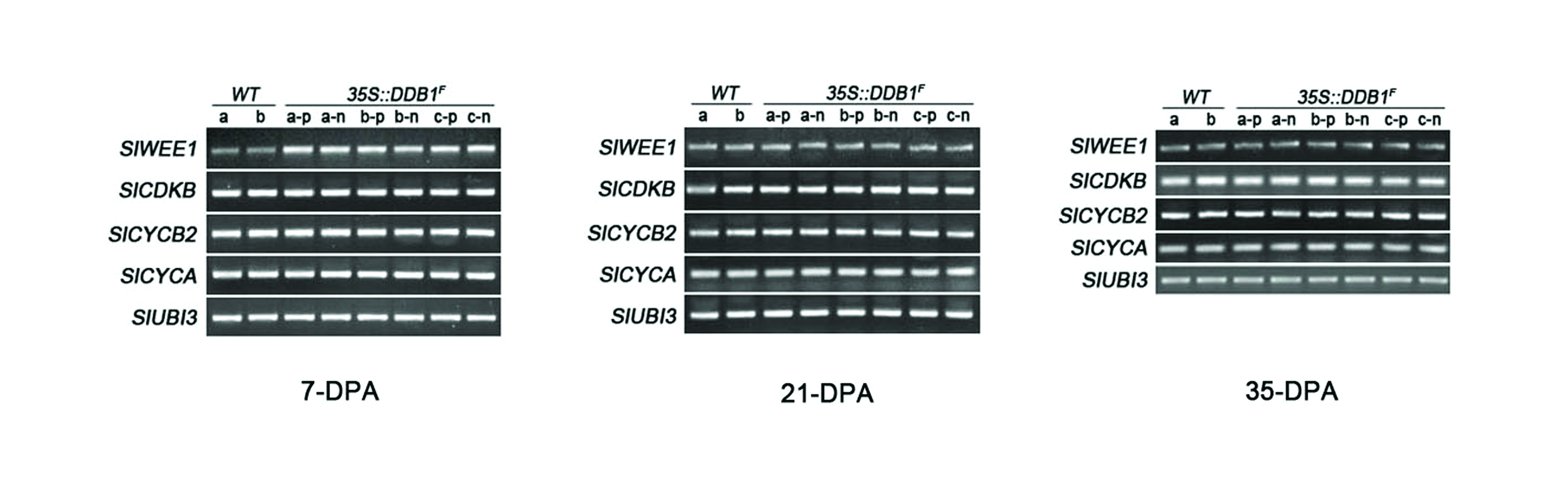

Supplement: Figure S2 — Semi-quantitative RT-PCR analysis of mRNA levels of SlWEE1, SlCDKB, SlCYCB2 and SlCYCA genes at different fruit development stages. Semi-quantitative RT-PCR analysis of mRNA levels of SlWEE1 gene and cell cycle controlling genes in 7-DPA, 21-DPA and 35-DPA fruits from WT Ailsa Craig (WT) and 35S::DDB1F T2 transgenic lines (a, b, c). p and n represent plants with or without transgene, respectively. Since the expression level of each gene was reduced with the fruit development, we used different PCR cycles at each stage. 7-DPA: 25 cycles; 21-DPA: 28 cycles; 35-DPA: 32 cycles. (TIF) [file pone.0042621.s002.tif]
